# Towards a Correlation between Structural, Magnetic, and Luminescence Properties of CeF_3_:Tb^3+^ Nanocrystals

**DOI:** 10.3390/ma13132980

**Published:** 2020-07-03

**Authors:** Cristina Bartha, Corina Secu, Elena Matei, Catalin Negrila, Aurel Leca, Mihail Secu

**Affiliations:** National Institute for Materials Physics, 077125 Magurele, Romania; cristina.bartha@infim.ro (C.B.); cesecu@infim.ro (C.S.); elena.matei@infim.ro (E.M.); cnegrila@infim.ro (C.N.); lec.aurel@gmail.com (A.L.)

**Keywords:** fluorides, nanocrystals, structure, magnetism, photoluminescence

## Abstract

In this study, we report on the structural, magnetic, and optical properties of Tb^3+^-doped CeF_3_ nanocrystals prepared via a polyol-assisted route, followed by calcination. X-ray diffraction analysis and electron microscopy investigations have shown the formation of a dominant Ce_0.75_F_3_ nanocrystalline phase (of about 99%), with a relatively uniform distribution of nanocrystals about 15 nm in size. Magnetization curves showed typical paramagnetic properties related to the presence of Ce^3+^ and Tb^3+^ ions. The magnetic susceptibility showed a weak inflexion at about 150 K, assigned to the cerium ions’ crystal field splitting. Under UV light excitation of the Ce^3+^ ions, we observed Tb^3+^ green luminescence with a quantum yield of about 20%.

## 1. Introduction

Rare-earth-doped nanofluorides are of great interest due to their high potential for application in various fields, e.g., lighting and displays, optical amplifiers, lasers, up-converters, and scintillators ([1,2,3] and references therein). Nanomaterials showing both luminescence and magnetic characteristics in particular are useful for a wide range of applications, such as multifunctional imaging and simultaneous diagnosis and therapy [4,5].

Among different inorganic fluorides, CeF_3_ is one of the most versatile due to its wide range of bulk properties, such as high thermal and chemical stability, high density and resistance to radiation, fast response time, low vibrational energies, etc., and chemical flexibility of the structure allowing high solubility of rare-earth dopant ions. Another major advantage of this compound is related to the large spectrum of possible preparation methods, allowing for its processing in various sizes and morphologies, down to the nanometer scale. The bulk CeF_3_ crystal has a hexagonal phase structure: a space group of *P*3*c*1 (*D*_3*d*_^4^) with lattice constants *a =* 0.713 nm and *c* = 0.729 nm [5]. It exhibits efficient UV absorption due to the allowed electric dipole 4f→5d transition of Ce^3+^ ions and energy transfer to other rare-earth activators, which is useful for white LED phosphor applications [6,7,8]. In particular, various synthesis methods have been proposed for Tb^3+^-doped CeF_3_ nanocrystals, and the photoluminescence properties and energy transfer mechanism have been extensively investigated [9,10,11,12]. A less-studied aspect is related to the magnetic properties, which are strongly correlated with its electronic structure, whereas for the rare-earth dopant ions, these are related to the unpaired electrons in the inner 4f sub-shell. Investigations of the magnetic behavior of CeF_3_ crystals are relatively limited and have been described within several models: crystal field theory [13], mixed-valent Ce^3+^-Ce^4+^ behavior [14], or super-exchange interaction of cerium ions [15]. Recent investigations of CeF_3_ nanodisks have revealed an anomalous behavior of the magnetic susceptibility at T ≅ 50 K, assigned to the processing-related defects and/or size effects [13,14,15,16]. 

The synthesis route may influence nanocrystals’ structure through synthesis-induced defects, but to date, there has been little focus on the structural refinement analysis, and the mixed valency behavior of the cerium ions has been overlooked [17]. These aspects are crucial to understanding the interaction mechanisms relevant for both optical and magnetic properties, and their improvement for possible applications. In addition, the high ratio of ions at the surface (with unsaturated coordination), and the periodicity loss on the surface, result in structural modifications and defects that may affect all the properties, and therefore, knowledge of the nanocrystals’ surface properties is highly relevant. The absolute novelty of this study derives from two aspects: (i) the complex structural analysis of terbium-doped cerium fluoride nanocrystals and its correlation with the influence of the material processing method, and (ii) the investigation of the specific magnetic behavior of the nanocrystals.

Herein, we report on the structural, magnetic, and optical properties of Tb^3+^-doped CeF_3_ nanocrystals prepared via a polyol assisted route. X-ray diffraction and electron microscopy techniques have been used for the structural and morphological characterization of the nanocrystals, whereas photoelectron spectroscopy was employed for the nanocrystals’ surface analysis. Magnetic and optical properties (e.g., photoluminescence, quantum yield) have been presented and discussed.

## 2. Materials and Methods 

### 2.1. Samples Preparation

For the preparation of Tb (8 mol%)-doped CeF_3_ nanocrystalline powders, we used the chemical precipitation technique at room temperature mediated by the ethylene glycol solvent, similar to [18]. The raw materials Ce(NO_3_)_3_ · 6H_2_O (99.5%), Tb(NO_3_)_3_ · xH_2_O (99.9%), NH_4_F (99.99%), and anhydrous ethylene glycol (99.8%) were used for the synthesis. Initially, a first solution was prepared by dissolving 0.102 g of NH_4_F into 10 mL of ethylene glycol. Then, 0.3261 g Ce(NO_3_)_3_ · 6H_2_O (99.5%) and 0.0358 g Tb(NO_3_)_3_ · xH_2_O (99.9%) were dissolved into 5 mL of ethylene glycol. This second solution was slowly dropped into the first one, and the mixture was stirred for 1 min at room temperature until a transparent colloidal solution had formed. At the end, the nanoparticles were isolated by centrifugation of the colloidal solution, washed to remove the remaining reagent, and dried at 80 °C in air. Further annealing of the CeF_3_:Tb powder was performed in open atmosphere for 1 h at 400 °C. 

### 2.2. Samples Characterization

For the X-ray diffraction (XRD) measurements, we used a BRUKER D8 ADVANCE (Billerica, Massachusetts, USA), type X-ray diffractometer. The XRD pattern was recorded in the 20 to 60° range with 0.05° step and 3 s integration time. For the phase composition analysis and crystal structure refinement, we used the Rietveld method and a dedicated software (MAUD) [19,20], and starting parameters from PDF 04-005-7362 (Ce_0.75_F_3_) and PDF 00-008-6551 (CeO_1.66_) files from the ICDD Powder Diffraction Files database [21]. The morphology of the samples was studied using a Zeiss MERLIN (Jena, Germany) Compact scanning electron microscope (SEM) with a GEMINI column, whereas for the chemical composition analysis, we used an EVO 50 XVP microscope from Zeiss equipped with an energy dispersive X-ray system (EDX) Quanta Bruker 200. For the magnetic properties’ characterization, we used a superconducting quantum interference device (SQUID), and we measured the sample magnetization dependence on both magnetic fields up to 3 T, and on temperatures ranging from 5 to 300 K. A powder sample (17 mg) of CeF_3_:Tb^3+^ was measured using a standard capsule, and its magnetic contribution (about 4%) was subtracted from the total magnetic signal. The temperature dependence of the magnetic susceptibility was measured under magnetic fields of 0.1 and 5 T applied perpendicularly to the sample. For the XPS measurements, we used a multianalysis SPECS system dedicated to surface analysis, equipped with a non-monochromatic source that provides a uniform X-Ray flux on the sample surface. The electron analyzer was a PHOIBOS150, with a 150 mm radius and a multichanneltron detector operating in large area mode and very low angular acceptance of 5° around the normal. The spectra of C1s, O1s, Ce3d, F1s, Tb3d, and Tb4d lines were recorded using a Pass Energy of 10 eV, while the extended spectrum was recorded using a Pass Energy of 50 eV. For the spectra fitting and analysis, we used the Spectral Data Processor v.2.3 software (XPS International, Marlborough, Massachusetts, USA) and Voigt functions. In order to minimize the contamination, the XPS spectra were recorded on freshly calcinated samples after having been stored for 24 h in the high vacuum chamber. For the photoluminescence (PL) spectra, we used a FluoroMax 4P spectrophotometer (HORIBA Jobin Yvon, Kyoto, Japan); the spectra were corrected for the spectral sensitivity of the experimental set-up. The chromaticity analysis and quantum yield characterization were performed using the Quanta-Phy accessory of the spectrophotometer”. 

## 3. Results and Discussion

### 3.1. Structural and Morphological Characterisation

**Figure 1:**
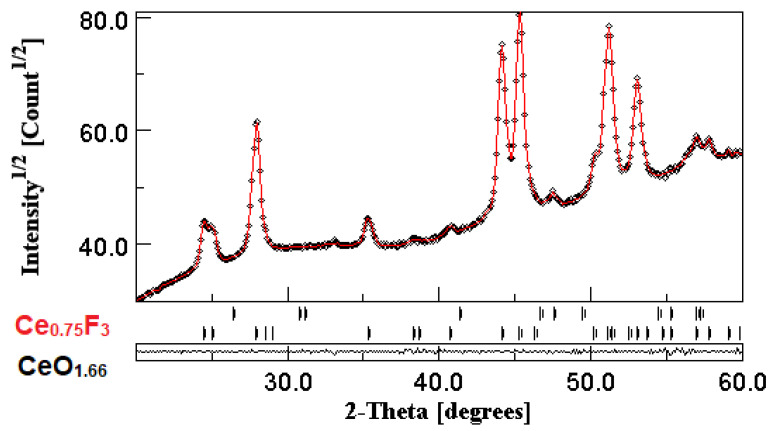
XRD pattern (black curve) and Rietveld refinement plot (red curve) of CeF_3_:Tb nanocrystalline powder calcinated at 400 °C, with the Bragg reflections for Ce_0.75_F_3_ and CeO_1.66_ crystalline phases indicated by vertical bars; the lower trace represents the difference curve between observed and calculated patterns.

The XRD pattern of the CeF_3_:Tb nanocrystalline powder calcinated at 400 °C is depicted in Figure 1; the results of the Rietveld analysis are presented in Figure 1 and Table 1 and Table 2. The analysis showed the presence of a dominant Ce_0.75_F_3_ crystalline phase (of about 98.7%), accompanied by a much smaller CeO_1.66_ crystalline fraction (of about 1.2%), both stable at room temperature. The formation of Ce_0.75_F_3_ as a dominant crystalline phase is the consequence of the synthesis and processing conditions that result in a material with defects and multiple fraction valencies of cerium ions [17]. The lattice parameters are very close to those from the ICDD database (Table 1), and the nanocrystallite’s size agrees very well with SEM microscopy data (see below).

From Table 2, it can be observed that the positions of the Ce^3+^ ions and their first neighbor (F_1_ ions) are slightly shifted compared to the reference. As the Tb^3+^ ions ionic radius (R = 1.095A) is smaller than that of Ce^3+^ ions (R = 1.196A), a substitutional incorporation is very likely; the photoluminescence spectra showed a crystal-field structure of the Tb^3+^ luminescence bands (see below). However, the XRD pattern analysis has shown small changes in the cell parameters (Table 1), and therefore, a partial interstitial incorporation of Tb^3+^ ions cannot be completely rejected. The microstrain values for both phases indicated different values of the distances between the crystallographic planes, which can be related to the lattice defects produced during the synthesis and lattice distortion induced by the Tb^3+^ ions’ incorporation into the Ce_0.75_F_3_ crystalline lattice.

**Figure 2:**
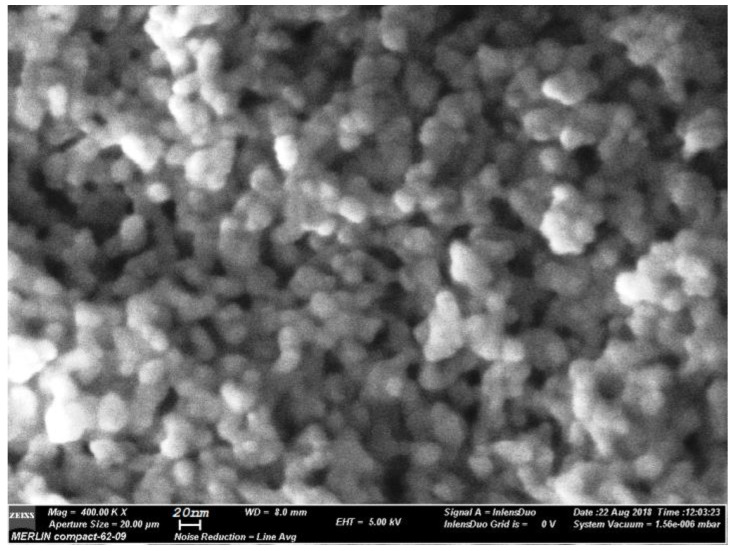
SEM image of CeF_3_:Tb nanocrystalline powder calcinated at 400 °C.

**Figure 3:**
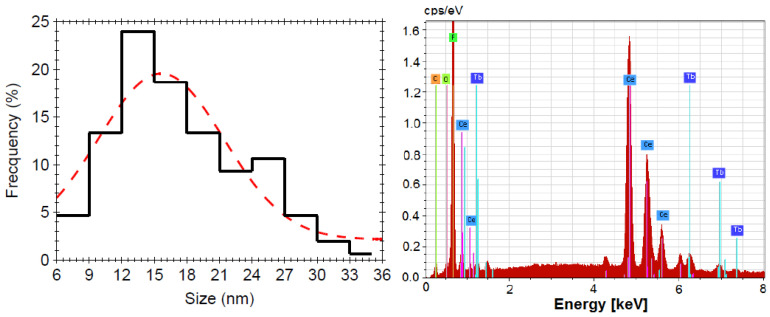
Nanocrystals size distribution (**left**) and the corresponding energy dispersive X-ray (EDX) analysis with the characteristic X-ray lines assignment (**right**); the dotted curve is just a guide for the eyes.

The SEM image analysis has shown a distribution of round-shaped nanoparticles of about 15 nm in size (Figure 2 and Figure 3). Chemical composition analysis resulting from the EDX spectra (Figure 3) has shown a relative agreement (within the experimental errors) between the atoms’ concentrations in the precursor chemicals and in the annealed samples: 14 at%(Ce), 79 at%(F), 1.5 at%(O), and 1.5 at%(Tb).

It can be seen that the F to Ce ratio (of about 5.5) is relatively close to the four expected for Ce_0.75_F_3_ phase (Table 1), but higher than the three expected for CeF_3_, indicating Ce^3+^ ion deficiency and lower Tb^3+^ ion concentration than expected; XRD analysis has shown Ce_0.75_F_3_ phase, i.e., Ce^3+^ ion deficiency. We suppose that the reason is related to the NH_4_F amount used for the synthesis, since it plays a critical role in controlling the final morphology and size of the product [11]. In the present case, for the stoichiometric NH_4_F amount generally used, we obtained spherical nanoparticles about 15 nm in size (Figure 2) with Ce^3+^ ion deficiency as resulting from the EDX and XRD analyses (Table 1 and Figure 3), this aspect having been overlooked by previous studies. The Ce^3+^ ion deficiency suggested that a higher NH_4_F amount is required to accomplish the reaction and the Tb^3+^-doping process. However, for higher NH_4_F content, different nanocrystals morphologies were obtained with hundreds of nm in size, accompanied by crystallinity and luminescence signal improvement [11]. Therefore, by using a stoichiometric NH_4_F amount, we obtained nanocrystals “with defects”, i.e., a Ce_0.75_F_3_ nanocrystalline phase with lower “effective” Tb^3+^ ions’ dopant concentration.

### 3.2. X-ray Photoelectron Spectroscopy (XPS) Characterization

For the investigation of the nanocrystals’ surface, we used the XPS technique, which is recognized as a valuable investigation tool for the chemistry associated to different bonds and related compounds ([22] and references therein).

**Figure 4:**
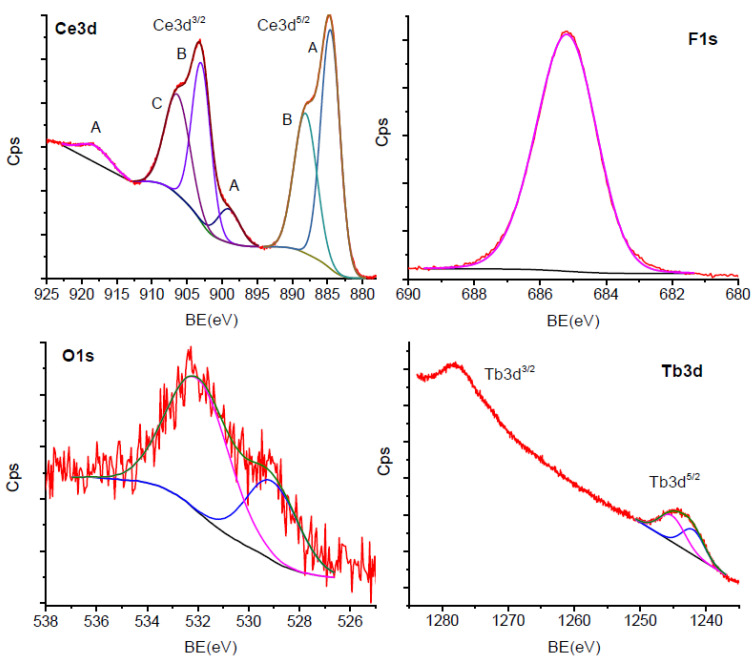
The XPS spectrum of the CeF_3_:Tb nanocrystalline powder calcinated at 400 °C.

Figure 4 shows different spectral lines of the XPS spectrum of CeF_3_:Tb nanocrystalline powder annealed at 400 °C. The region between 880 and 925 eV was assigned to the Ce3d line of the Ce^3+^ ion species, and the energies indicate Ce-F bonds [23]. The XPS spectrum is composed of several convoluted peaks that correspond to the Ce 3d^5/2^ and 3d^3/2^ lines, and each of them is split into two lines due to the multiplet splitting effect arising from the two possible states after photoionization: 4f^1^ and 4f^2^. The 884.57 and 902.97 eV peaks were due to the 4f^2^ final state, whereas the others at 888.12 and 906.38 eV were due to the 4f^1^ final state. The 899.05 and 918.11 eV peaks were assigned to the “loss” type peaks due to the energy transfer from the F2p orbitals to the conduction band orbitals of Ce (4d,5d,6s) [24,25]. The CeF_3_ nanocrystalline phase was revealed by the strong single F1s peak at 685.20 eV, in connection to the Ce 3d peaks. The oxygen species’ characteristic peaks are observable in the energy region at around 530–532 eV. The 529.07 eV peak was assigned to the oxidized metal, whereas the 531.99 eV peak was assigned to the adsorbed species and carbon bonds; low signal of the oxygen indicates low oxygen concentration. The oxidized metal species were observed in LaF_3_ nanoparticles within a nanometric layer at their surfaces [18] and are related to the calcination process. Regarding the Tb^3+^-ion dopant, its characteristic spectrum in the 4d region between 140 and 160 eV showed a complex multiplet splitting (not presented), which is difficult to analyze. However, the values of the energy bonds and the spectrum shape attribute the observed features to Tb^3+^ ion species. In the 3d region, the Tb3d^3/2^ peak overlaps with the Ce3p^1/2^ peak, and the two 1241.96 and 1245.19 eV energy peaks were assigned to the Tb3d^5/2^ multiplet splitting, corresponding to the 3d^9^4f^9^ and 3d^10^4f^8^ final states. As the bond energies are slightly higher than for corresponding metal or oxides [26,27], and fluorine electronegativity is higher than for oxides, the peaks were assigned to the Tb-F bonds, due to the Tb^3+^ incorporation in the CeF_3_ matrix.

### 3.3. Magnetic Properties

**Figure 5:**
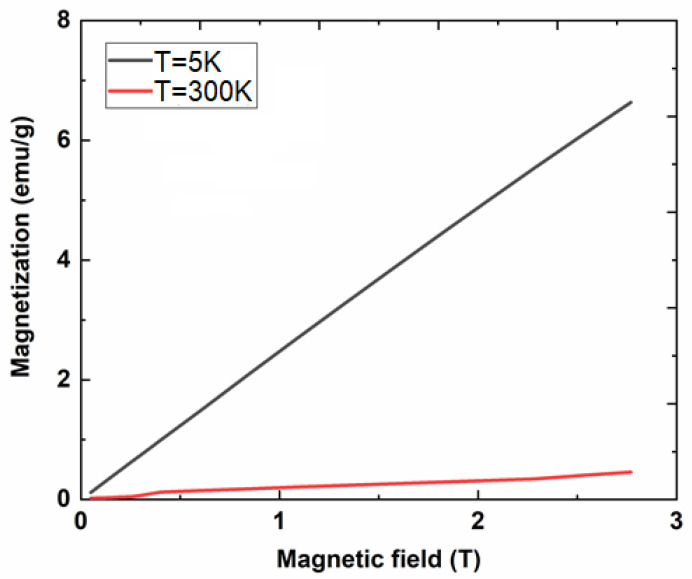
Magnetization vs. magnetic field dependence measured at T = 300 K and T = 5 K recorded on CeF_3_:Tb nanocrystalline powder calcinated at 400 °C.

The paramagnetic properties of the CeF_3_:Tb^3+^ nanocrystalline powder calcinated at 400 °C are confirmed by the magnetization vs. magnetic field dependencies (at low and high temperatures) of the magnetization curves (Figure 5). 

**Figure 6:**
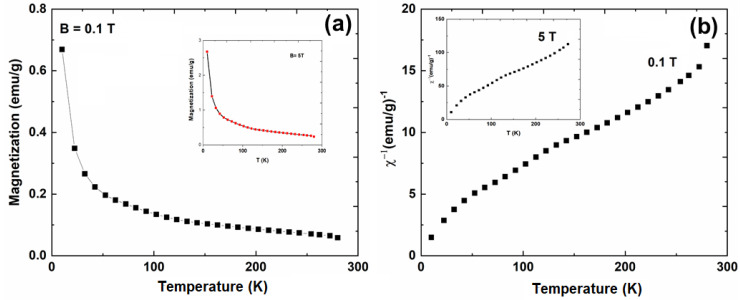
Temperature dependencies of the magnetization at an applied magnetic field of 5 T (inset) and 0.1 T, respectively, (**a**) and the inverse magnetic susceptibility at a perpendicularly applied magnetic field of 5 T (in inset) and 0.1 T, respectively (**b**).

Temperature dependence of both magnetization and the inverse magnetic susceptibility (computed from the experimental data) are depicted in Figure 6. In both cases (for applied magnetic fields of 0.1 and 5 T), the magnetic susceptibility shows a typical paramagnetic behavior with small inflection at around 150 K that can be attributed to the Ce^3+^ ions’ crystal field splitting. Tb^3+^ contribution is very weak considering the small percentage of Ce^3+^ substitution. It is known that cerium is a Kramer’s ion, having an odd number of 4f electrons, and the maximum possible splitting of the ^2^F_5/2_ state is in three doublets. When Ce^3+^ ions are placed in a hexagonal symmetry, the maximum splitting of levels will occur [28]. Energy splitting calculations showed that, for a hexagonal crystal field in cerium, the doublets were excited to 30 and 150 K above the ground state [29]. These values are comparable to those reported at 89 and 206 K for Ce^3+^ ions in hexagonal yttrium [30].

### 3.4. Photoluminescence Properties

The photoluminescence properties and energy transfer mechanism for Tb^3+^-doped CeF_3_ have been extensively investigated in CeF_3_ [9,10,11,12], and therefore, we do not address them in detail here. The energy levels of Tb^3+^ allow an efficient energy transfer (ET) from the 5d-4f emission of the Ce^3+^ ions, resulting in high light output (Figure 6—left). Under 250 nm UV light pumping, Ce^3+^ ions are excited from ^7^F_7/2,5/2_ ground state levels to the 4f5d excited energy levels. Then, a radiative energy transfer to the Tb^3+^ excited levels occurs, followed by radiative deexcitation from the green emitting levels of Tb^3+^ (^5^D_4_→^7^F_j_; J = 5, 4, 3)—Figure 6. The maximal ET rate is about 89% for 7.5 mol% Tb^3+^-doping [5], or 79.7% for 10mol% Tb^3+^-doping [10]. In the present case, we computed the ET efficiency (η_ETE_) from Ce^3+^ to the Tb^3+^ activator ions using the Ce^3+^ luminescence signal intensities in the absence (I_0_) and presence (I) of Tb^3+^-activator ions [31]: “η_ETE_ = 1−I/I_0_”, and we obtained an ET efficiency value of about 92%.

**Figure 7:**
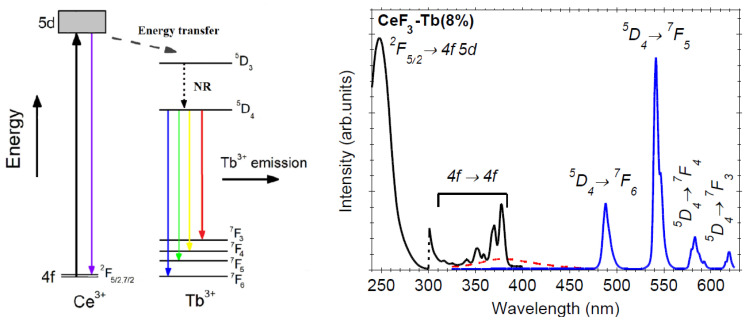
Energy level scheme of the Ce^3+^ and Tb^3+^ ions (**left**) and the photoluminescence excited at 250 nm with the excitation spectrum of the 545 nm luminescence recorded on CeF_3_:Tb nanocrystalline powder calcinated at 400 °C (**right**); photoluminescence of undoped CeF_3_ nanocrystalline sample is shown for comparison (dotted curve).

The luminescence and excitation spectra recorded on undoped and Tb-doped CeF_3_ nanocrystalline powders calcinated at 400 °C are depicted in Figure 7. Under 250 nm UV light excitation, the undoped nanoparticles showed weak 375 nm luminescence assigned to the 5d-4f radiative deexcitation (Figure 7—dotted curve) [9]. This luminescence cannot be seen in the doped nanocrystal sample, where we observed only the Tb^3+^-related luminescence peaks at 542, 585, and 620 nm (^5^D_4_→^7^F_j_; J = 5, 4, 3). The PL bands showed a crystal field structure associated to Ce^3+^ substitution by the Tb^3+^ in the CeF_3_ crystalline lattice. The excitation spectrum of the green 545 nm Tb^3+^-luminescence showed the characteristic 4f-4f transitions of the Tb^3+^ ions, accompanied by a broad and intense peak at 250 nm assigned to the Ce^3+^ transitions ^7^F_7/2,5/2_ to 4f5d excited states. 

**Figure 8:**
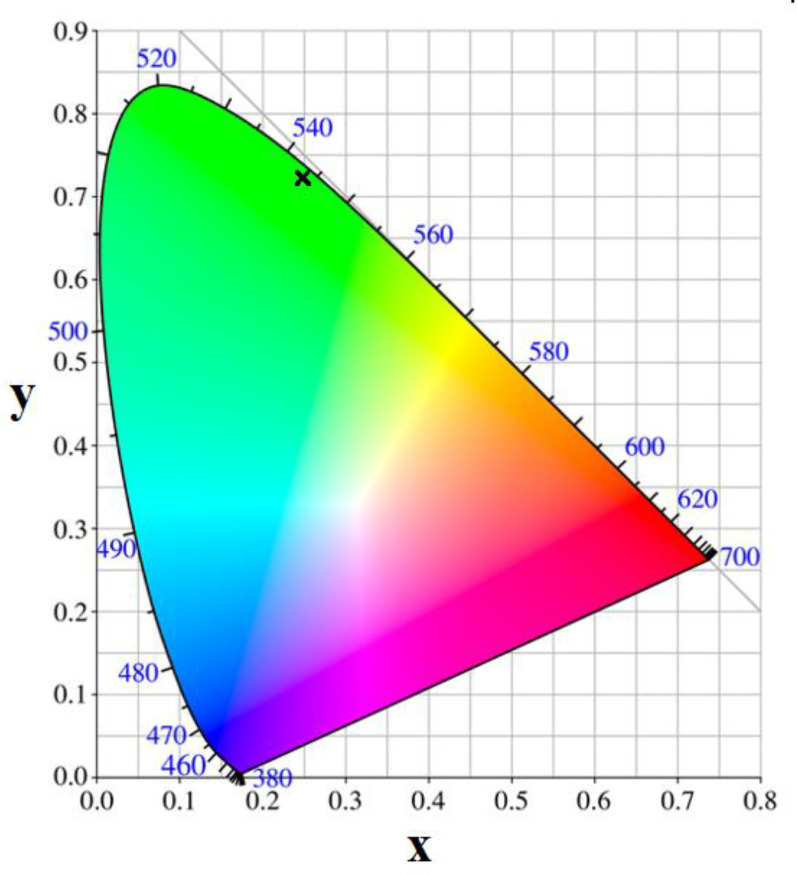
The chromaticity coordinates of the Commission Internationale de l’Eclairage (CIE) chromaticity diagram of CeF_3_:Tb nanocrystalline powder calcinated at 400 °C.

Figure 8 shows the Commission Internationale de l’Eclairage (CIE) chromaticity diagram of CeF_3_:Tb^3+^ nanocrystalline powder with the coordinates x = 0.252 and y = 0.725 in the region of green light characteristic to the Tb^3+^ luminescence region. In the present case, the quantum yield (QY) of the Tb^3+^ ions green-related luminescence is about 20% (for 250 nm excitation wavelength) and is smaller than for YAG:Ce phosphor nanoparticles (of about 130 to 270 nm size), where a QY of 70–72% was reported [32]. On the other hand, the QY is influenced by the nanocrystals’ size: the QY of Ca_3_Sc_2_Si_3_O_12_:Ce^3+^ nanopowders is about 50% and increases to 70–72% for microparticles [33].

## 4. Conclusions

In this study, Tb^3+^-doped CeF_3_ nanocrystals showing both magnetic and photoluminescence properties were prepared at room temperature via a polyol mediated route followed by calcination. 

X-ray diffraction analysis has shown a dominant Ce_0.75_F_3_ nanocrystalline phase (of about 99%), in which Tb^3+^ ions are substitutionally and interstitially incorporated, accompanied by traces of CeO_1.66_ (of about 1%). Electron microscopy investigations have shown a relatively uniform distribution of nanocrystals about 15 nm in size. X-ray photoelectron spectroscopy has evidenced the presence of an oxidized metal layer at the nanocrystals’ surface due to the calcination process. Magnetization curves showed typical paramagnetic properties related to the Ce^3+^ and Tb^3+^ ions. The magnetic susceptibility behavior for low and high applied magnetic fields (0.1 T and 5 T, respectively) highlighted a small inflection around 150 K, due to the crystal field splitting related to the cerium ions. Under UV light excitation, an efficient energy transfer from the Ce^3+^ to Tb^3+^ ions resulted in a strong Tb^3+^ green luminescence with a quantum yield of about 20%.

The combination of fluorescent alongside magnetism-integrated functions of multifunctional nanoparticles could lead to new opportunities in nano-bio related ***applications***.

## Figures and Tables

**Table 1:** Rietveld refinement results for Tb^3+^doped CeF_3_ nanocrystalline powders calcinated at 400 °C.

Crystalline Phase	Cerium Fluoride (Ce_0.75_F_3_)	Cerium Oxide (CeO_1.66_)	R Factors (%)
Weight fraction (%)	98.798 ± 0.001	1.2017 ± 0.002	
Crystal size (nm)	13.985(2)		
Crystal system	Hexagonal	Cubic	R_wp_ (%) = 2.994 R_B_ (%) = 2.358 R_exp._ = 1.015 χ^2^ = 2.049
Space group	P-3c1	Fm-3m	
Calculated Unit Cell (Å)	a = b = 7.1053(3) c = 7.2614(1)	a = 5.415(1)(2)	
Cell_angle_alpha Cell_angle_beta Cell_angle_gamma	90° 90° 120°	90° 90° 90°	
Unit Cell according PDF (Å) (ref. [21])	a = b = 7.1 c = 7.27	a = b = 5.4112(10)	
Cell Volume (Å^3^)	317.38 (3)	158.45(1)	
Microstrain	1.7 × 10 ^−5^ ± 0.002	2.3x10 ^−6^ ± 0.001	

**Table 2:** Atomic site occupancy for Tb^3+^doped CeF_3_ nanocrystalline powders.

Atoms	Ce_0.75_F_3_ (calc.)	Ce_0.75_F_3_ (theor.)	Ce_0.75_F_3_ (calc.)	Ce_0.75_F_3_ (theor.)	Ce_0.75_F_3_ (calc.)	Ce_0.75_F_3_ (theor.)	Wyckoff Site	Atom Site Occupancy (theor/calc)
**x**			**y**		**z**			
F_1_	0.3862(3)	0.356	0.3610(4)	0.328	0.0712(3)	0.096	12g	1/0.8393(5)
Ce	0.3394(5)	0.3333	0	0	0.2504(1)	0.25	6f	0.75/0.7503(2)
F_3_	0.3334(2)	0.3333	0.6671(5)	0.6666	0.2267(3)	0.167	4d	1/0.9851(5)
F_4_	0	0	0	0	0.2497(2)	0.25	2a	1/0.9877(4)
